# Hafnium isotope constraints on the nature of the mantle beneath the Southern Lau basin (SW Pacific)

**DOI:** 10.1038/s41598-020-74565-0

**Published:** 2020-10-15

**Authors:** Quanshu Yan, Susanne Straub, Paterno Castillo, Haitao Zhang, Liyan Tian, Xuefa Shi

**Affiliations:** 1 Key Laboratory of Marine Sedimentology and Environmental Geology, First Institute of Oceanography, Ministry of Natural Resources, Qingdao, 266061 China; 2 Laboratory for Marine Geology, Qingdao National Laboratory for Marine Science and Technology, Qingdao, 266061 China; 3 Lamont-Doherty Earth Observatory, Columbia University, Palisades, NY 10964 USA; 4 Scripps Institution of Oceanography, University of California, San Diego, La Jolla, CA 92093 USA; 5 Institute of Deep-Sea Science and Engineering, Chinese Academy of Sciences, Sanya, 572000 China

**Keywords:** Ocean sciences, Solid Earth sciences

## Abstract

New Hf isotope data provide new insights into the nature of the mantle beneath the southern Lau basin, adding new constraints on the displacement process of the Pacific mid-ocean ridge basalt (MORB)-type mantle by the Indian MORB-type mantle. The Hf isotopic ratios (^176^Hf/^177^Hf) of submarine lavas from the eastern Lau spreading center (ELSC) range from 0.283194 (εHf = 14.92) to 0.283212 (εHf = 15.54), with an average value of 0.283199 (εHf = 15.11) whereas those from the Valu Fa ridge (VFR) vary from 0.283221 (εHf = 15.88) to 0.283200 (εHf = 15.14), with an average of 0.283214 (15.61), indicating that ELSC lavas have a slightly more radiogenic Hf isotopic composition than VFR lavas. In contrast to the results from previous studies, the new Hf analyses combined with previous Nd isotope data clearly show that both VFR and ELSC have the distinct Hf–Nd isotope composition of the so-called DUPAL isotopic anomaly in the Indian MORB-type mantle. The DUPAL isotopic signature at VFR demonstrates for the first time that the inflow of the Indian MORB-type mantle has reached the southern tip of tectonic propagation in the southern Lau basin.

## Introduction

The Lau basin-Tonga arc system, similar to the Mariana arc/backarc basin system, is an intra-oceanic convergent margin with little influence from continental crust materials. Therefore, the system has been considered as an ideal place for testing the plate tectonic and seafloor spreading hypotheses^1,2^, as well as for modeling the magmatic processes intimately associated with plate subduction^3–18^. Additionally, hthis system is ideally suited for investigating the relationship between subduction input and arc/backarc output^19,20^.

Previous studies had shown that lavas from Lau backarc spreading axes contain variable contributions from the subducted slab^13,15,17,21–23^. Along-axis (nearly latitudinal) compositional variations may be due to variable amounts of slab-derived fluids, different lengths and ways of transport of such fluids, and the diversity of amount and type of sediment entering the ‘ambient mantle’ above the subducting plate^15,17,23,24^. Moreover, the original Pacific mid-ocean ridge basalt (MORB)-type mantle (PMM) in the Lau basin-Tonga arc system has been influenced by the inflow of Indian MORB-type mantle , possibly following the 'docking' of Ontong-Java plateau and/or the collision of the Samoan plume with the northern part of the Lau basin-Tonga arc system^5,18,25–32^. The scope of the Indian MORB-type mantle influence in the Lau backarc basin, however, is still in debate^22,33,34^.

The Indian MORB mantle source between Bouvet Island and the Australian-Antarctic Discordance has a distinct DUPAL isotopic anomaly, characterized by positive delta ^208^Pb/^204^Pb and delta ^207^Pb/^204^Pb, high ^87^Sr/^86^Sr, low ^206^Pb/^204^Pb (old Pb) and a wide range of delta εHf values^35^. In contrast, the Indian MORB-type mantle in SW Pacific, although also having the DUPAL signature, is different as it has a characteristically high, Pacific MORB-like ^206^Pb/^204^Pb (relatively young Pb). Therefore, the occurrence of an “Indian MORB mantle”in SW Pacific^22^ is confusing because this mantle did not come from the Indian Ocean asthenosphere^36^; its origin is more likely due to processes operating in the SW Pacific region, such as melt depletion of dispersed Samoa plume material^37^ or process operating on some other DUPAL sources^22,33,38^. Thus, in this study, we refer to the Indian MORB-type mantle in the Lau basin-Tonga arc system as the DUPAL-like Indian MORB-type mantle.

With the gradual southward propagation of the backarc rift zones, the DUPAL-like Indian MORB-type mantle mantle is thought to have wedged into and replaced the pre-existing original Pacific MORB mantle beneath the Tonga arc and Lau backarc basin^6,22,39^. The influx of fluids containing components from the slab (subduction components) into the mantle wedge also added compositional variation to the ambient mantle^13,22,24,40–42^. Therefore, precisely identifying the composition of ambient mantle is a key to the clarification of the distribution patterns, as well as the displacement process, of the original Pacific MORB mantle and inflowing DUPAL-like Indian MORB-type mantle.

Studies involving traditional Sr, Nd and Pb isotopic ratios, combined with trace element data, have shown the simultaneously presence of the Pacific MORB mantle and Dupal-like Indian MORB-type mantle domains in the Lau basin-Tonga arc system, but the precise boundary between the two remains unclear^8–10,14,15,17,23^. A major reason is that these geochemical tracers are also influenced by the addition of subduction components. Relative to the elements Sr, Pb and even the less-fluid mobile Nd, Hf is least mobile in fluids derived from the subducting slab^22,43–46^, although it may be mobile particularly if the fluids contain partial melts^47–49^. Therefore, after constraining the characteristics and amount of subduction components in arc and backarc lavas, the application of combined Hf and Nd isotopic systematics has been widely acclaimed to be the best tracer of the nature and composition of ambient mantle beneath volcanic arc and backarc regions^22,24,40–43,49–51^. Because the DUPAL-like Indian MORB-type mantle is characterized by higher εHf and lower εNd than the Pacific MORB mantle^18,22,41,43,48,52,53^, a combined Nd-Hf isotopic investigation may allow for a better tracing of these mantle domains in the Lau basin-Tonga arc system.

To date, Hf isotopic data for lavas from backarc spreading axes in the Lau basin-Tonga arc system are still scarce^22,51^. This study presents new Hf isotopic data for the eastern Lau spreading center (ELSC) and Valu Fa ridge (VFR) lavas from the southern Lau basin in order to better constrain its ambient mantle composition^40^ and to refine the boundary between the Pacific MORB mantle and DUPAL-like Indian MORB-type mantle in the region. The new data provide some clues for the complex backarc spreading dynamics and inflow mechanism of DUPAL-like Indian MORB-type mantle accompanying the opening of Lau backarc basin. For the purpose of comparison, we also compile published Hf-Pb-Sr–Nd isotope data for Lau basin, Tonga ridge, Lau ridge and Samoa volcanic chain from the GEOROC (Geochemistry of Rocks of the Oceans and Continents) database (https://georoc.mpch-mainz.gwdg.de/georoc/Start.asp).

**Figure 1:**
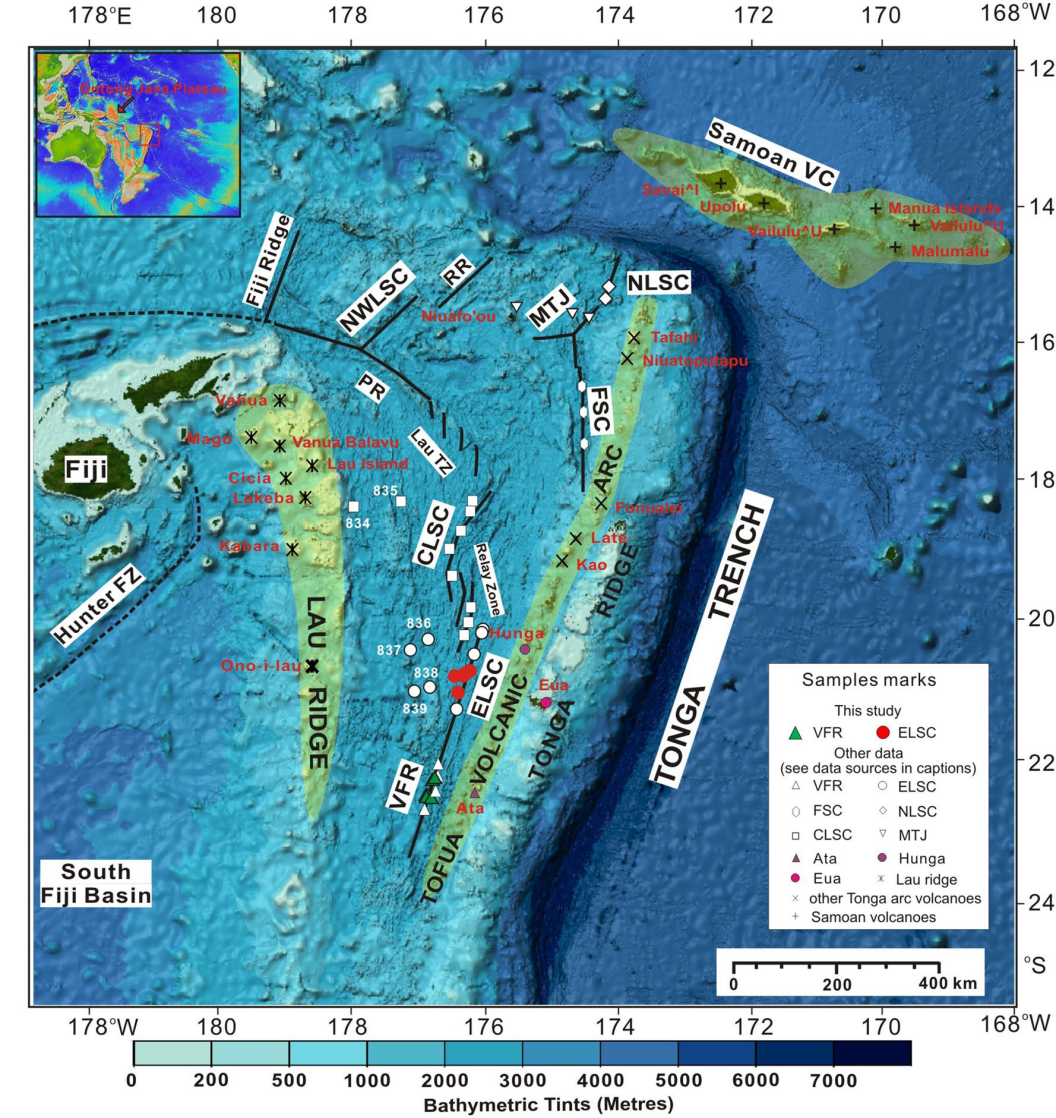
Geological sketch map of the Lau basin, showing major spreading centers and geographical features, sub-aerial and Samoa volcanic regions, and volcanoes (yellow triangles). Based on geological characteristics, seven regions are defined: Valu Fa Ridge (VFR), Eastern Lau Spreading Center (ELSC), Central Lau Spreading Center (CLSC), Northwestern Lau Spreading Center (NWLSC), Northeastern Lau Spreading Center (NELSC), Mangatolu Triple Junction (MTJ), and Folualei Spreading Center (FSC). Other major features include Peggy Ridge (PR), Rochambeau Ridge (RR), Hunter Fracture Zone (Hunter FZ), Samoan Volcanic Chain (Samoan VC), and Lau Extensional Transform Zone (Lau TZ). The locations of samples used in this study and in previous studies are also shown. Sources of other data are as follows: ELSC data including samples from ODP sites 836, 837 and 839 are from references^17,22,39,51^; CLSC data including samples from ODP sites 834 and 835 are from references^22,39,51^; VFR data are from references^17,22,24^; FSC data are from^22,62^; NLSC from references^18,22,30^; MTJ data are from references^22,30,62^. Tofua active arc data from Late, Kao, Ata and Hunga Ha’apai islands, and Tonga ridge (an inactive remanant forearc with relatively old (20–46 Ma) volcanics) data from Eua island are from references^22,27,51^; Lau ridge are from references^22,51^; and Samoan volcanoes from GEOROC database—https://georoc.mpch-mainz.gwdg.de/georoc/Start.asp). Insert shows the location of Lau basin on a regional scale. This map was created using GeomapAPP, Version 3.6.10 (https ://www.geomapapp.org/).

Lau Basin, located between the active Tonga Arc to the east and the Lau Ridge (a remnant volcanic arc) to the west in SW Pacific (Fig. 1), is an active intra-oceanic backarc basin. It has a mean depth of 2300 m, a length of 1000 km and is V-shaped, with a width of approximately 450 km in the north (15°S) that narrows to about 200 km in the south (25°S)^1,11,54–56^. Due to the integrated effects of Pacific plate subduction beneath the Australian plate and the docking of Ontong Java plateau plus the collision with the Samoan plume in the north, the Lau basin has undergone a relatively complex backarc spreading history. The basin can be roughly divided into three broad regions defined by major tectonic features: (1) Mangatolu triple junction to the northeast of Niua Fo’ou Island, (2) Central Lau Spreading Centre (CLSC) (including Peggy ridge) and (3) ELSC and its propagating rift tip—Valu Fa ridge^11,40,57^. Lau Basin opened from north to south starting at 6 Ma in two stages^11,54^. The first stage is a ‘Basin and Range’ style of rifting that is followed by seafloor spreading through rift propagation at about 5 Ma, and formed the ancient Lau Basin. The second is an extensional stage (induced by southward rift propagation) since 2 Ma that formed the current Lau Basin containing CLSC, ELSC and Valu Fa ridge. The ELSC has a full spreading rate of 95 mm/year (north) to 73 mm/year (south) at the Valu Fa ridge , which is only about 40 km from the Ata Island in the Tofua arc^54^ (Fig. 1). Detailed geophysical descriptions of the Lau-Tonga system have been presented in a number of previous studies^2,58–61^.

Samples analyzed in this study were collected in situ from ELSC and Valu Fa ridge in the southern Lau Basin using an underwater remotely operated vehicle equipped with manipulator arms and cameras during the 19th Chinese global expedition of the *R/V Dayangyihao* in 2007. Twelve samples consisting of three glass and nine whole rocks (six from Valu Fa ridge and six from ELSC) (red stars in Fig. 1) were selected for Hf isotopic analysis in this study. Five of the sites are on spreading axes (L-1, L-7, L-8, L-9, and L-11) and the rest are on ridge flanks (L-2, L-3, L-4, L-5, L-6, L-10, and L-12). These samples have been described previously in Yan et al.^17^.

## Results

**Figure 2:**
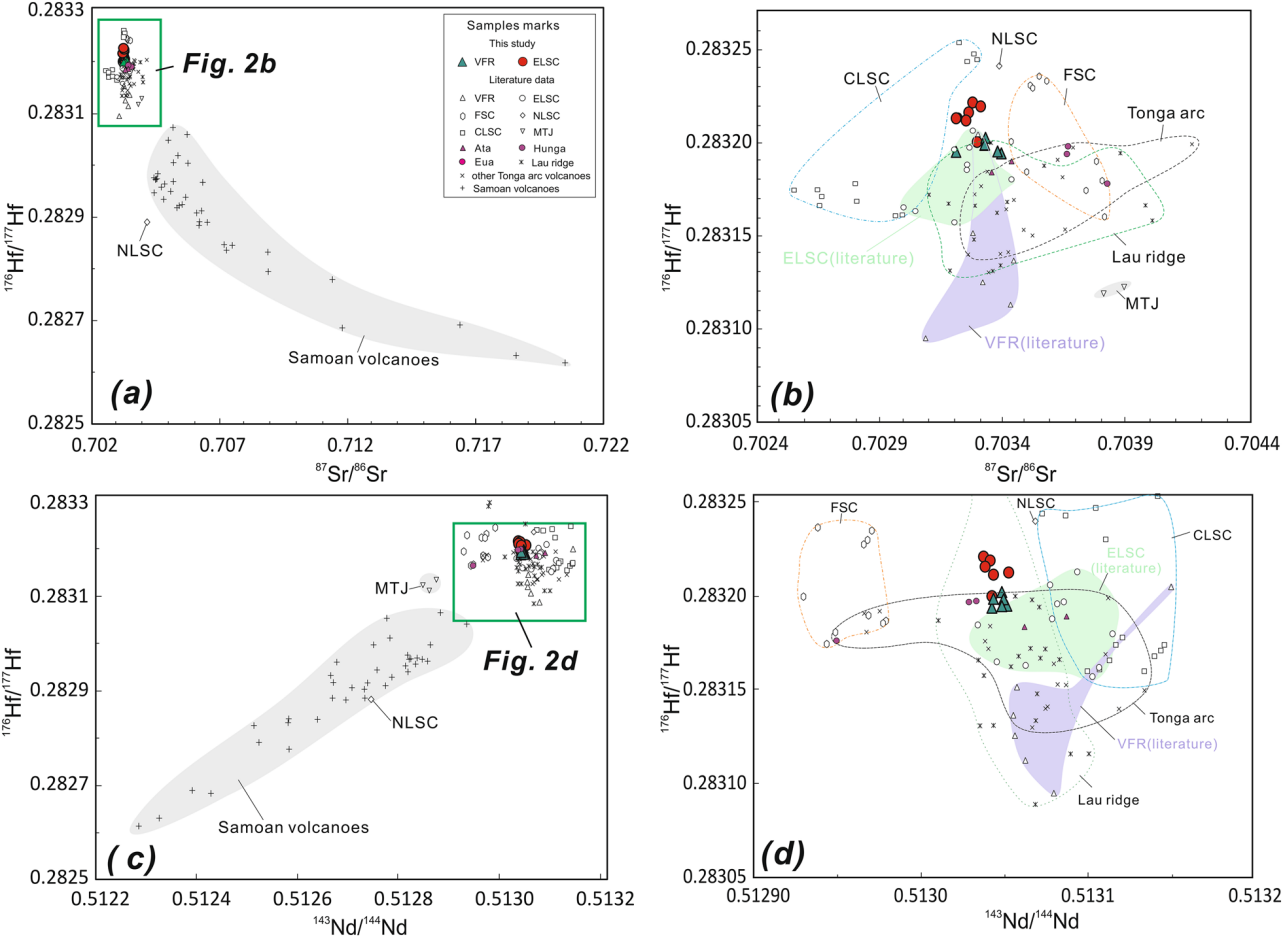
Plots of ^177^Hf/^176^Hf versus ^87^Sr/^86^Sr (**a**,**b**) and ^143^Nd/^144^Nd (**c**,**d**) for ELSC and Valu Fa ridge (VFR) lavas from the southern Lau basin. Data are shown in expanded scale in (**b**) and (**d**) to show the new results in more detail. Symbols and data sources are the same as Fig. 1.

**Figure 3:**
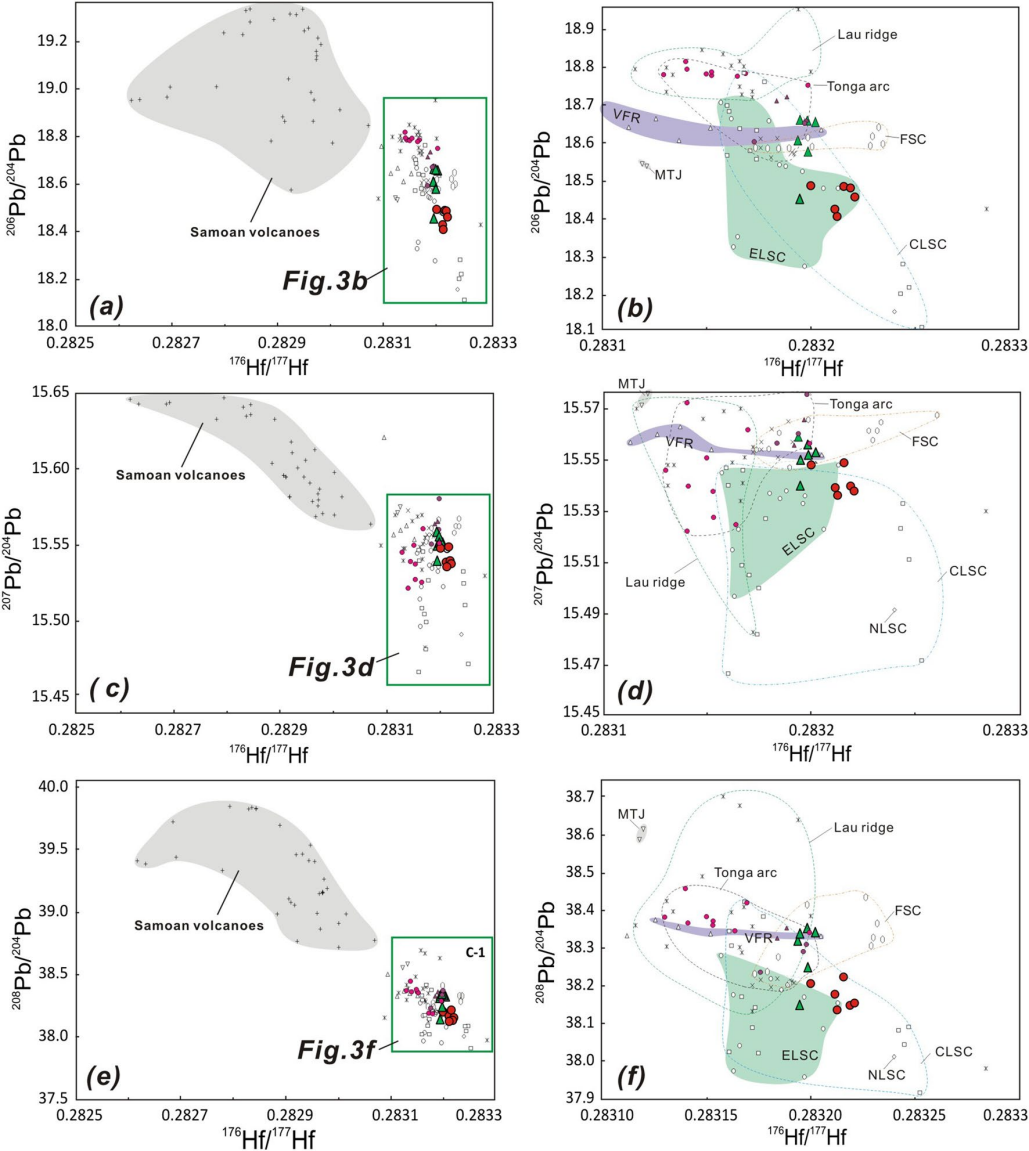
Plots of ^177^Hf/^176^Hf versus ^206^Pb/^204^Pb (**a**,**b**), ^207^Pb/^204^Pb (**c**,**d**) and ^208^Pb/^204^Pb (**e**,**f**) for ELSC and Valu Fa ridge (VFR) lavas from the southern Lau basin. Symbols and data sources are the same as in Fig. 1.

**Figure 4:**
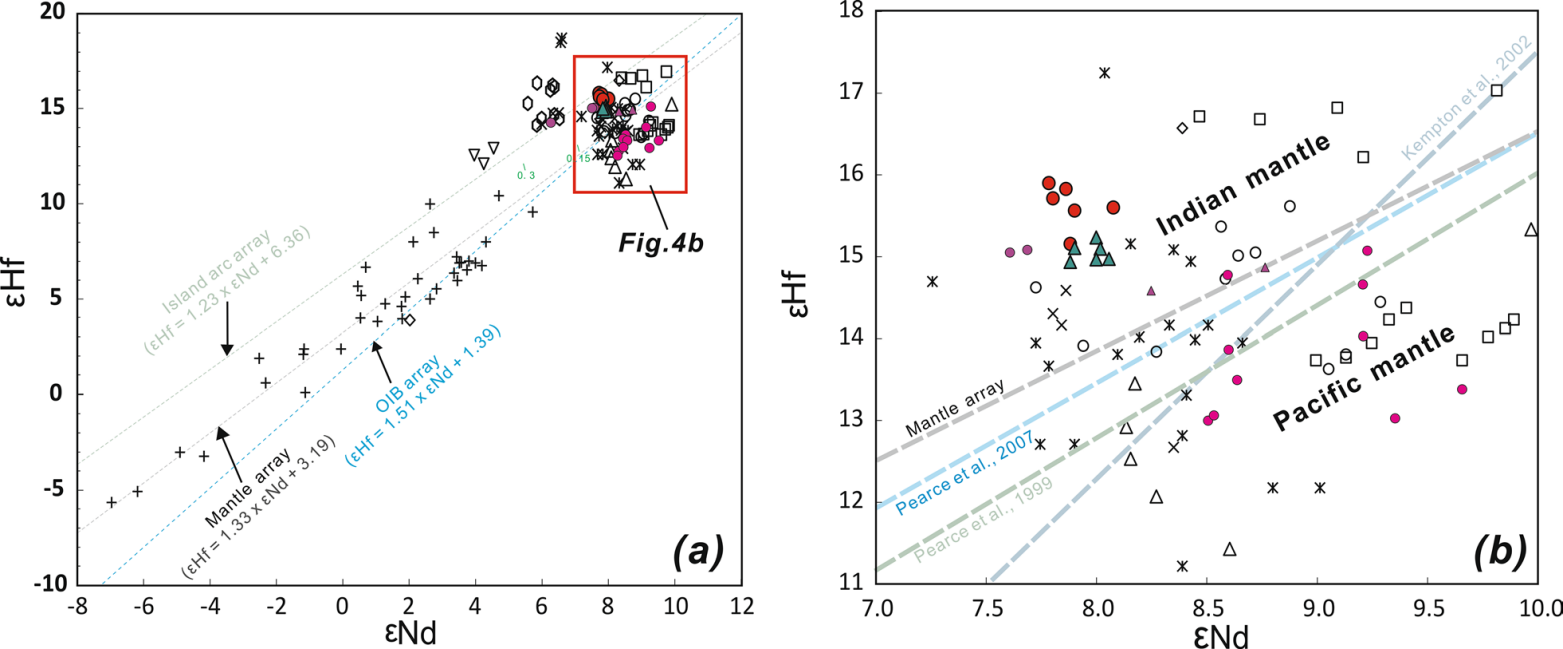
Plots of εNd versus εHf for ELSC and Valu Fa ridge (VFR) lavas from the southern Lau basin. In (**a**), the mantle array (εHf = 1.33 × εNd + 3.19)^65^, oceanic island basalt (OIB) array (εHf = 1.51 × εNd + 1.39)^52^ and island arc array (εHf = 1.23 × εNd + 6.36)^53^ are shown for reference. In (**b**), the pertinent equation to separate the Indian MORB mantle from the Pacific MORB mantle in different regions are as follows: εHf = 1.522 × εNd + 1.26for the Southwest Pacific^22^, εHf = 1.6 × εNd for Mariana region^43^, and εHf = 2.65 × εNd − 8.94 for the Australian-Antarctic Discordance (AAD)^50^. Symbols and data sources are the same as in Fig. 1.

The Hf isotopic compositions of the samples analyzed are presented in Supplementary Dataset Table S1 and shown graphically in Figs. 2 and 3. The new data have a narrow range of ^176^Hf/^177^Hf composition (0.283194–0.283202 (< 1 εHf unit) for the Valu Fa ridge and 0.283200–0.283221 (< 1 εHf unit) for ELSC, consistent with their homogeneous ^143^Nd/^144^Nd composition (0.513042–0.512051 (< 1 εNd unit) for Valu Fa ridge and 0.513037–0.513052 (< 1 εNd unit) for ELSC). Compared to ELSC lavas, Valu Fa ridge lavas have slightly higher or more radiogenic ^176^Hf/^177^Hf ratios. The new data for both Valu Fa ridge and ELSC lavas are within the isotopic range of the lavas from the entire Lau basin-Tonga arc system, but in general, they have higher ^176^Hf/^177^Hf for given ^87^Sr/^86^Sr, ^143^Nd/^144^Nd and Pb isotope ratios compared to those published Valu Fa ridge and ELSC data. In general, the new Valu Fa ridge and ELSC data are close to those for lavas from Ata and Hunga islands in the main volcanic (Tofua) arc, but are different for lavas from Eua island in the forearc (or remnant/older arc) to the east of Tofua arc (Figs. 1, 2, 3, 4) . In the εNd versus εHf plot, the new Valu Fa ridge and ELSC data fall in the Indian MORB-type mantle field above the mantle array^65^ and oceanic island basalt (OIB) array^52^ (Fig. 4). Although not shown, they also plot in the Indian MORB-type mantle field in the ^208^Pb/^204^Pb versus ^206^Pb/^204^Pb discrimination diagrams being used to distinguish the Indian MORB-type mantle from the Pacific MORB mantle by Pearce et al.^22^ (for the Southwest Pacific), Pearce et al.^43^ (for Mariana region), and Kempton et al.^50^ (for the Australian-Antarctic Discordance).

## Discussion

The combined Hf–Nd isotope systematics has been widely used to delineate mantle domains beneath the vast and geologically complex western Pacific trench-arc-backarc (Mariana and Lau basin-Tonga arc) systems^22,24,40–43,50,51^. It was in these systems that the two types of mantle sources, Pacific MORB mantle and DUPAL-like Indian MORB-type mantle, were identified^17,22,42^. However, the boundary between the two mantle domains based on Hf–Nd isotope systematics in the Lau Basin is not entirely clear, and this is due to the limited amount of data^22^.

Yan et al.^17^ showed that most of the same Valu Fa ridge and ELSC lavas analyzed in this study lie in the overlapping Pacific MORB mantle and Dupal-like Indian MORB-type mantle fields and also partly in the field for Tonga arc lavas, which have been affected by subduction components, based on their Sr–Nd–Pb isotopic composition. Thus, these isotopic tracers do not unambiguously identify the boundary between Pacific MORB mantle and DUPAL-like Indian MORB-type mantle in the ambient mantle. Previously, Staudigel et al.^33^ and Pearce et al.^22^ proposed that the inflowing Dupal-like Indian MORB-type mantle derived from south Pacific isotopic and thermal anomaly^33^ or other regions^34,66^ has wedged into ELSC, but since the Valu Fa ridge still has a Pacific MORB mantle signature similar to the southern Tonga arc, it was suggested that the displacement of the Pacific MORB mantle by the DUPAL-like Indian MORB-type mantle did not reach the southern tip of the Valu Fa ridge. However, our new data show that lavas from Ata and Hunga islands in the Tofua volcanic arc are close to those for Valu Fa ridge and ELSC, but is different from those for lavas from Eua island in the forearc (or older/remnant arc) (Figs. 1–4), implying that the nature of mantle source beneath the southern Tofua arc is similar to that beneath the Valu Fa ridge and ELSC and different from that beneath Eua island in the Tonga ridge (forearc or remnant arc).

Below, we use the new Hf isotopic data, combined with previously published Nd isotopic data^17^, to further evaluate the replacement of the Pacific MORB mantle by the inflowing DUPAL-like Indian MORB-type mantle in the ambient mantle beneath the southern Lau Basin.

### The effect of subduction components on the combined Hf–Nd isotope systematics

Available Hf isotope data for the Lau basin-Tonga arc system lavas cover a moderate compositional range^22^. The new Hf–Nd isotope data for ELSC and Valu Fa ridge lavas clearly show that their lavas plot within the IMM field (Fig. 4), inconsistent with the results from previous studies^17,22,51^. However, the new data may not represent the true ambient mantle Hf–Nd isotope composition because of the negative Hf anomalies in the normalized trace element concentrations of the lavas (see Fig. 5 in Yan et al.^17^). Arc lavas from a mantle wedge containing subduction components are generally depleted relative to other trace elements in high field-strength elements, particularly Nb but also Hf^47–49^, and, thus, the negative Hf concentration anomalies imply that the ambient mantle beneath ELSC and Valu Fa ridge had been affected by the subduction components as well. Thus, it is necessary to constrain the subduction components influence on the measured Hf–Nd isotope composition of the lavas first before it can be successfully used to discriminate the Indian MORB-type mantle from the Pacific MORB mantle in the southern Lau basin^18,22,41–43,50^. Accordingly, we evaluate whether the Nd and Hf concentrations and isotopic compositions of Valu Fa ridge and ELSC lavas had been influenced by the subduction components or not, and if they were, what is the combined Hf–Nd isotope composition of their mantle source after such influence has been removed from the measured values.

**Figure 5:**
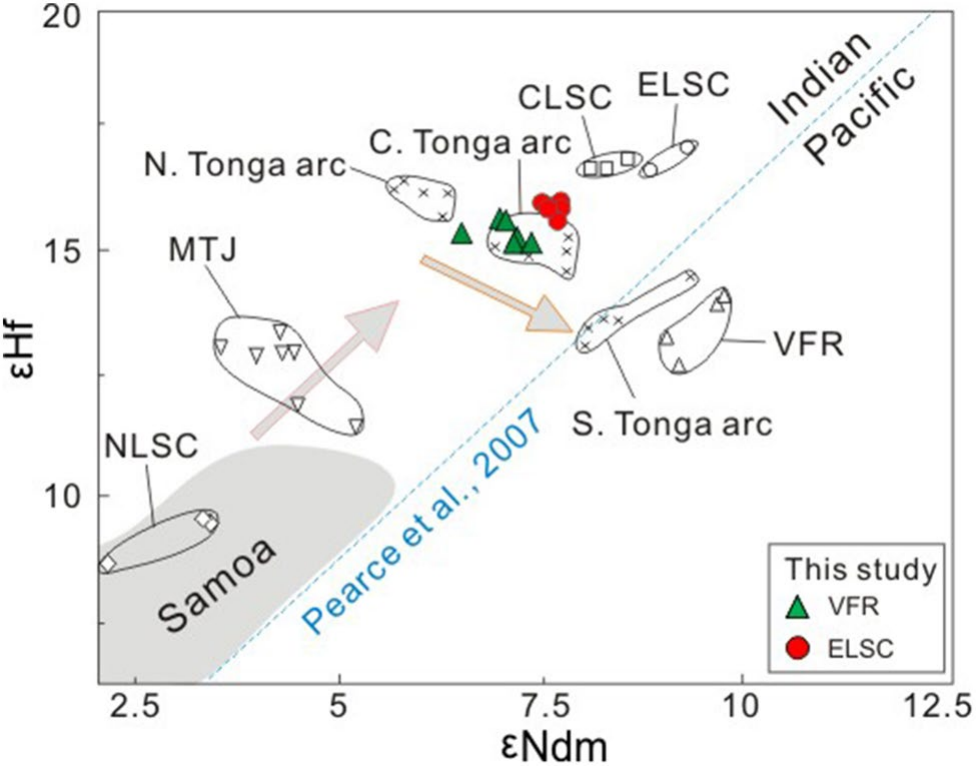
Plot of εHf versus εNdm for ELSC and Valu Fa ridge (VFR) lavas from the southern Lau basin^22^. εNd_m_ values refer to the εNd values of underlying mantle after correcting for the effect of subduction input (Supplementary Dataset Table S1). Symbols and data sources are the same as in Fig. 1.

A way to evaluate the influence of subduction components in the Hf–Nd isotope composition of the Valu Fa ridge and ELSC lavas is through the method of Woodhead et al.^41,48,49^. These authors claimed that the Hf and Nd isotopic compositions of backarc basin lavas may have been affected by solute-rich fluids containing partial melts of subducted sediments. In order to get the true composition of the ambient mantle beneath backarc basins, the influence of subduction components in backarc lavas should be first clarified and ruled out^40,41^. However, when we applied their filtering criteria (i.e., samples with Th/Ta < 3 and Ba/Nb < 7^41^), all Valu Fa ridge and ELSC lavas show evidence of the subduction components. In fact, almost all Lau basin lavas have Ba/Nb ratios > 7, implying the presence of subduction components beneath the entire Lau basin^15–17,23,30,67^. Lavas from southern Mariana arc system also do not pass the same filtering criteria and, thus, Ribeiro et al.^42^ calculated the original isotopic composition of the pre-subduction mantle instead. The isotopic compositions of the pre-subduction mantle source of the Valu Fa ridge and ELSC lavas following Ribeiro et al.’s method^42^ (i.e., filtering the Hf–Nd isotopic ratios of lavas from their subduction influence) are also listed in Supplementary Dataset Table S1. Results show that 5% of slab fluids were added to the ambient mantle; after deducting such subduction contribution, the corrected εNd* and εHf* values for the Valu Fa ridge and ELSC lavas range from 8.7–11.6 and 16.1–19.1, respectively (Supplementary Dataset Table S1). Significantly, when these corrected data are plotted in the εHf versus εNd_m_ (the Nd isotopes of ambient mantle, see its calculating formula^22^ in Supplementary Dataset Table S1) diagram (Fig. 5), they still lie on the the Indian MORB-type mantle side of the Indian MORB-type mantle—the Pacific MORB mantle boundary of Pearce et al.^22^. Thus, the addition of a small of amount (< 5%) of slab fluids to the ambient mantle does not significantly affect the combined Hf–Nd isotope systematics, which indicates that both the Valu Fa ridge and ELSC lavas most likely came from a DUPAL-like Indian MORB-type mantle source.

**Figure 6:**
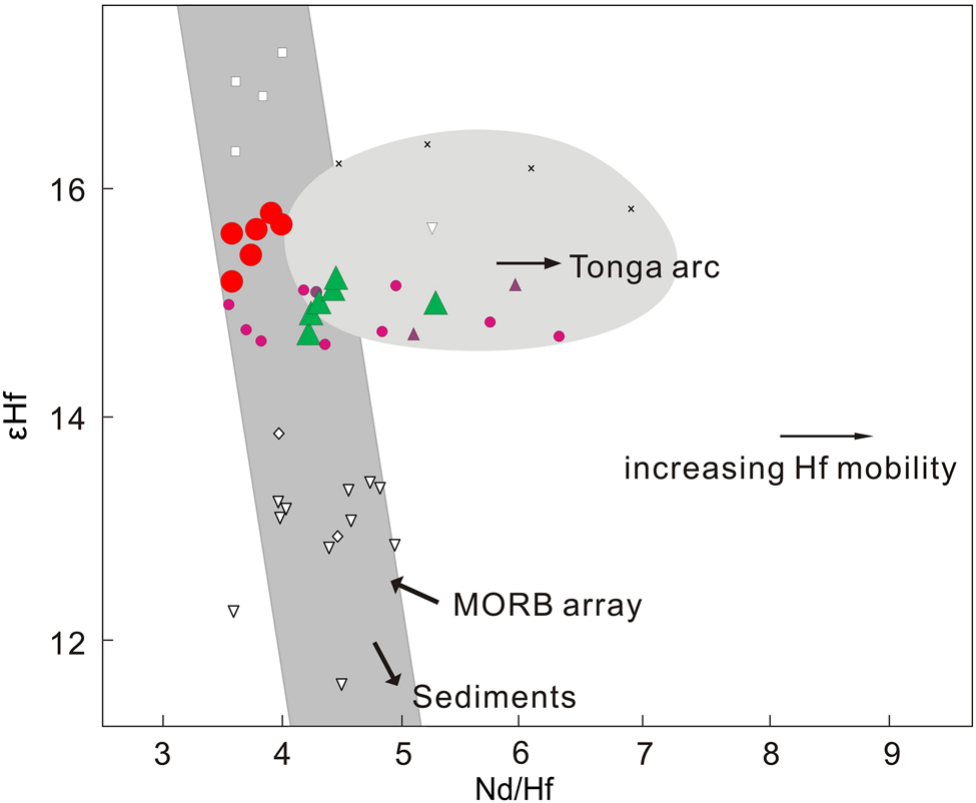
Plot of εHf versus Nd/Hf for ELSC and Valu Fa ridge (VFR) lavas from the southern Lau basin, to constrain Hf mobility in the Lau basin-Tonga arc system^22^. Symbols and data sources are the same as in Fig. 1.

Another way to evaluate the influence of subduction components in the Valu Fa ridge and ELSC lavas is through the use of the εHf versus Nd/Hf diagram^22^ (Fig. 6). The diagram shows that almost all our samples except for one Valu Fa ridge lava plot within the MORB array, which is distinct from the field for Tonga arc lavas that have been affected by the subduction components and, thus, have higher and variable Nd/Hf ratios for given εHf^22^. Therefore, the ^176^Hf/^177^Hf of the Valu Fa ridge and ELSC lavas were not influenced by the subduction components, implying that they basically represent the Hf isotopic composition of the ambient mantle.

To evaluate the influence of subduction components on the Nd concentration and isotopic composition in the lavas, we calculated their ΔNd values (proportions of Nd in the mantle wedge from the subduction addition), and ΔεNd_P/I_ (the displacement of εNd from the boundary between the Indian MORB-type mantle and Pacific MORB mantle) using the detailed calculations by Pearce et al.^22,43^. Results show that the ΔNd values of the Valu Fa ridge lavas vary from 0.195 to 0.441, and those of ELSC lavas range from 0.005 to 0.077 (Supplementary Dataset Table S1), suggesting that the Nd content in both the Valu Fa ridge and ELSC lavas received contributions from the subduction components, as also shown by previous studies^15,17,22,23,67^. However, the ΔεNd_P/I_ values for the Valu Fa ridge and ELSC lavas are above zero, with VFR lavas ranging from 1.28 to 1.53, and ELSC lavas ranging from 1.58 to 2.15 (Supplementary Dataset Table S1). The positive values indicate that the lavas are coming from a DUPAL-like Indian MORB-type mantle source, which is inconsistent with previous studies claiming that the Valu Fa ridge lavas just tap a Pacific MORB mantle source^22,24^. In summary, ΔεNd_P/I_ and ΔNd values (Supplementary Dataset Table S1) indicate that both ELSC and Valu Fa ridge lavas tap a DUPAL-like Indian MORB-type mantle source as well.

In summary, the above modeling results indicate that the ambient mantle beneath both ELSC and Valu Fa ridge had been affected by the subduction components, and this is better shown by the Nd isotope composition of the lavas. After correcting for such an effect, however, the combined Nd-Hf isotope data indicate a DUPAL-like Indian MORB-type mantle ambient mantle beneath ELSC and Valu Fa ridge. Thus, the addition of subduction components has a negligible effect on the Hf isotope composition. A possibility is that Hf, as a high field-strength element, is indeed immobile in subduction fluids, unless there is partial melting of the subducted sediment^47–49^. There is a consensus that Th/Nb or Th/Ta can be used as indicators of partial melting of sediment, although the geochemical behavior of Nb and Ta hosted in stable rutile in the subducted slab is still in debate^47–49^. The Th/Ta ratios of the Valu Fa ridge lavas range up to 5.5, with an average value of 4.0, and the only sample with Th/Ta < 3 is L-6 (2.8; Supplementary Dataset Table S1). In contrast, the Th/Ta ratios of ELSC lavas are all < 3, and ranging from 0.2 to 1.8, with average value of 1.2 (Supplementary Dataset Table S1). Thus, according to the criterion of Woodhead et al^41^, all Valu Fa ridge lavas have sediment contributions, except for sample L-6, which together with ELSC lavas have no sediment melt contribution. Significantly, however, the new Valu Fa ridge and ELSC data show a homogeneous Hf (and Nd) isotope composition (Figs. 2, 4, 6). Such a Hf and Nd isotope homogeneity suggests that the sediment contribution does not affect the compositional homogeneity of Hf isotopic ratios of these lavas, and is consistent with the idea that compared to isotopic tracers (e.g., Pb, Sr and to a certain extent Nd) that are affected by the subduction components, the element Hf is immobile (Fig. 6). In other words, the Hf isotopic ratios of Lau basin lavas are insensitive to the influence of slab components derived from the subducting plate^22^. Hence, the Hf and Nd isotopic composition of the Valu Fa ridge lavas, together with ELSC lavas, come from the DUPAL-like Indian MORB-type mantle mantle.

### Implications of the Indian MORB mantle beneath the Valu Fa ridge in the southern Lau basin

Pacific MORB mantle is ubiquitous in the whole Tonga-Lau ridge region before Lau back-arc spreading, u as all older volcanic rocks collected from Lau relict ridge and Tonga ridge until now didn’t tap the Indian MORB-type mantle (Figs. 2–4), implying that the Indian MORB-type mantle more likely are later stage exotic mantle. Previous studies have shown that in the Lau basin backarc region, a series of southward to southwestward propagating spreading rift axes, which accommodate crustal extension within the Pacific lithosphere^11,54^, have enabled the DUPAL-like Indian MORB-type mantle mantle to advect into the region underlain by a Pacific MORB-type mantle^22,39,51^. The fact that the Valu Fa ridge lavas have the Hf isotopic composition of the DUPAL-like Indian MORB-type mantle mantle already identified in the Northern Lau Spreading Center, Mangatolu triple junction, CLSC backarc spreading regions and northern and central Tonga arc implies that the DUPAL-like Indian MORB-type mantle mantle is flowing into the tip of propagating rift in the southern Lau basin. In turn, this indicates that mantle and tectonic processes are occurring contemporaneously during the opening of the Lau basin. Data in this study also clearly show that inflowing DUPAL-like Indian MORB-type mantle mantle has also reached the mantle beneath Hunga and Ata islands in the southern Tofua volcanic arc (Figs. 2–4). However, it appeared that the Indian MORB-type mantle did not reach the Eua island in the Tonga ridge (remnant or older forearc), because existing evidences for those relatively old volcanics with ages of about 20–46 Ma on the island (e.g., Eua island) just tapped the Pacific MORB mantle did not definitely support the idea that the mantle beneath Tonga ridge have been replaced by Indian-type mantle^22^. In fact, due to the inactive feature (no active volcanism) for these islands in remnant or older forearc, it is reasonable that there is no mantle processes (e.g., mantle emplacement) beneath these regions.

**Figure 7:**
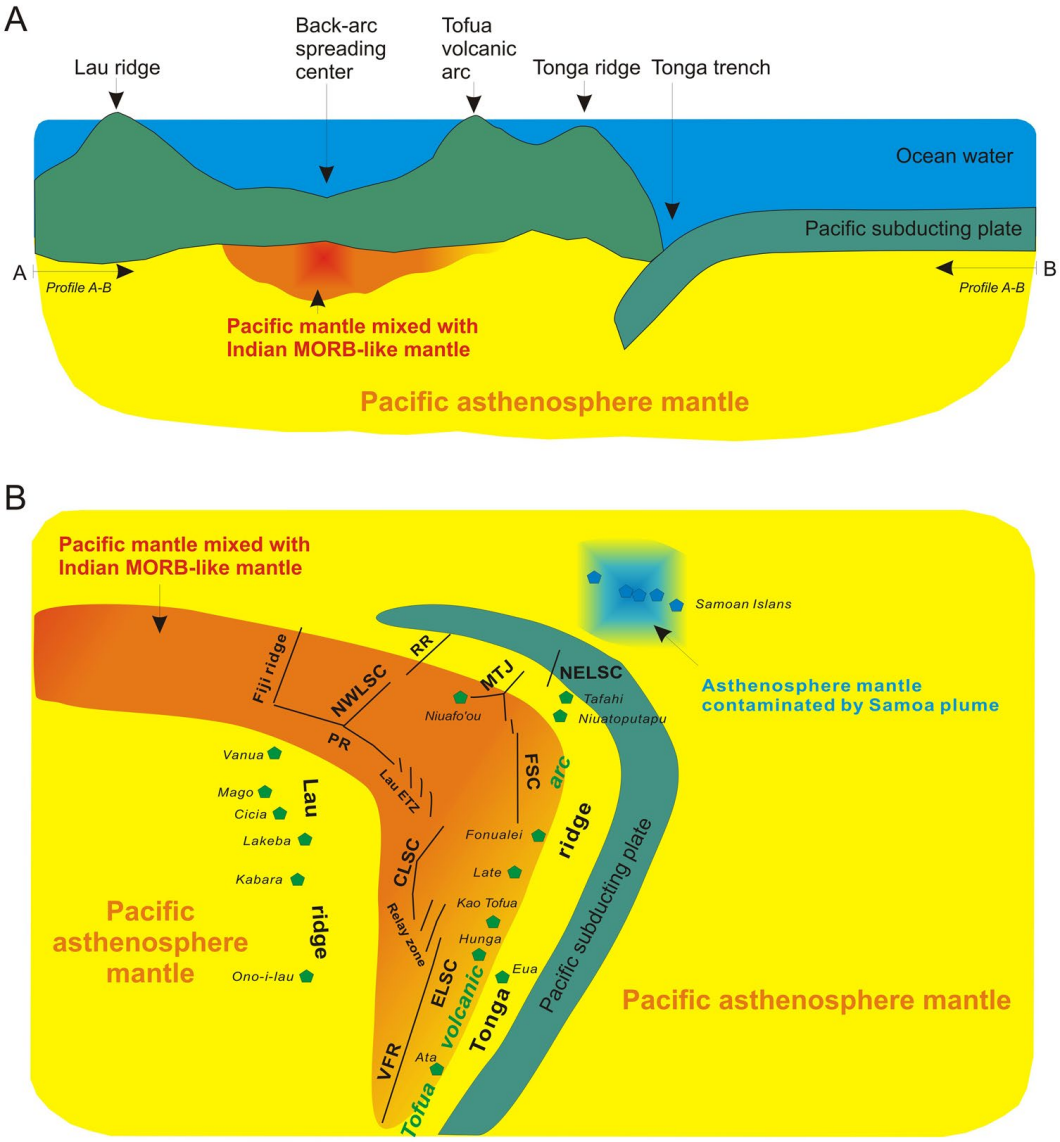
A cartoon model for the possible spatial distribution of the Indian MORB mantle beneath the Lau basin. (**A**) Pacific asthenosphere mantle is mixed with DUPAL-like Indian MORB mantle showing its longitudinal distribution beneath the back-arc spreading center. Profile A-B is at the depth of this mixed asthenosphere underlying the lithosphere. (**B**) The possible spatial distribution of asthenosphere mantle at the depth of Profile A-B. The pacific asthenosphere mantle components beneath the NWLSC, CLSC, ELSC, Valu Fa ridge and Tofua volcanic arc have been replaced by the DUPAL-like Indian MORB mantle, whereas Lau ridge and Tonga ridge are still maintaining the properties of pacific mantle asthenosphere. The asthenosphere mantle contaminated by Samoa plume is shown at the northeast of Lau basin. The colour gradient from red to yellow represents the influence strength of the Indian MORB mantle.

We developed a cartoon model for the possible spatial distribution of DUPAL-like Indian MORB-type mantle beneath the Lau basin, and proposed the replacement extent of the Pacific MORB mantle by the DUPAL-like Indian MORB-type mantle (Fig. 7). In the proposed tectonic scenario, the asthenosphere mantle partial melting process beneath Lau back-arc spreading centers provides a possibility that the migrating DUPAL-like Indian MORB-type mantle flows come into the melting zone and mix with the preexisting Pacific MORB mantle. With the gradual opening of Lau basin southwards, DUPAL-like Indian MORB-type mantle flows gradually propagate from the northwestern to the southern Lau basin. So far the southernmost position they have reached is at the southern part of the Valu Fa ridge. Meanwhile, the asthenosphere mantle beneath the Tofua volcanic arc which is proximal to back-arc spreading centers (e.g., CLSC, ELSC and Valu Fa ridge) has been contaminated (or replaced) by DUPAL-likee Indian MORB-type mantle. The pacific asthenosphere mantle components beneath the Northwest Lau Spreading Center, CLSC, ELSC, Valu Fa ridge and Tofua volcanic arc have been replaced by the Indian MORB-type mantle, whereas Lau ridge and Tonga ridge are still maintaining the properties of pacific mantle asthenosphere.

## Conclusion

In this study, we present new Hf isotope data that provide new insights into the mantle dynamics and tectonic processes in the southern Lau basin.

1. The Hf isotopic ratios (^176^Hf/^177^Hf) of submarine lavas from the ELSC and Valu Fa ridge range from 0.283194 (εHf = 14.92) to 0.283212 (εHf = 15.54), with the average value of 0.283199 (εHf = 15.11), and those from the Valu Fa ridge vary from 0.283200 (εHf = 15.14) to 0.283221(εHf = 15.88), with the average of 0.283214 (15.61).
2. In contrast to those of previous studies, results of this study clearly show that the Valu Fa ridge and ELSC lavas are relatively homogeneous and have a DUPAL-like Indian MORB-type mantle signature.
3. A key outcome of this study is that a DUPAL-like Indian MORB-type mantle source is already flowing into the southerly propagating Valu Fa ridge in the southern Lau basin, indicating contemporaneous nature of mantle dynamic and lithospheric tectonic processes in the Lau basin -Tonga arc system.

## Methods

Hafnium isotope ratios of the 12 samples were obtained at the Lamont Doherty Earth Observatory of Columbia University. Two whole rock samples (L-7 and L-11) were leached prior to digestion as replicates. For this purpose, sample powders were soaked in double-distilled 8 N HNO_3_ for one hour at ~ 95℃. The leached samples were centrifuged and the leachates were discarded. Centrifuging was repeated twice with double-distilled water. Residues were transferred into Teflon beakers, and dried down prior to routine sample digestion.

For each sample, about 200–250 mg of powder (about 200–250 mg) was digested using a 3:1 HF (ca. 27 N) + HNO_3_ (ca. 16 N) mixed acid, and several times re-digested with HNO_3_, 6 N HCL and finally a mixture of 6 N HCL + 0.06 N HF prior to dissolution in 3 N HCL. The chemical separation of Hf was achieved with the single, 1 ml column filled with Eichrom Ln resin (100–150 μm) following the procedure after Münker et al.^63^. Prior to loading onto the column, all Fe was reduced by adding about 0.5 ml 1 M ascorbic acid. After loading, all matrix elements such as Fe, REEs and Ti are washed out prior to Hf extraction in a mixture of 6 N HCl + 0.2 N HF.

Hafnium isotope ratios were measured using a ThermoFisher Neptune Plus multi-collector ICP-MS also at the Lamont Doherty Earth Observatory of Columbia University. Instrument performance was monitored by multiple measurements of an in-house Hf Spex standard (^176^Hf/^177^Hf = 0.28216) that was analyzed alternately with the samples. The Hf Ames standard is intercalibrated to the JMC-475 standard with the same ^176^Hf/^177^Hf ratio of 0.282160^64^ to which all measured ^176^Hf/^177^Hf ratios were normalized. Sample solutions were constantly monitored for levels of Lu and Yb, and ^176^Hf/^177^Hf ratios were only accepted when the ^176^Yb and ^176^Lu contribution to the ^176^Hf signal was < 0.6%, and thus negligible. Internal measurement errors are between 7–17 ppm (2σ). Replicate measurements of the Hf Spex standard give an external reproducibility of ^176^Hf/^177^Hf of 0.282156 ± 0.000005 (35 ppm, 2σ, n = 8) and ^176^Hf/^177^Hf of 0.282148 ± 0.000003 (21 ppm, 2σ, n = 25) during the two days of data collection in May and August 2013, respectively. For each batch, international standard BCR-2 was concurrently dissolved and analyzed as unknown and gave ^176^Hf/^177^Hf of 0.282865 (2σ = 8 ppm) and ^176^Hf/^177^Hf of 0.282869 (2σ = 15 ppm), respectively.

## Supplementary information

Supplementary Information.

## Data Availability

All data are reported in the Supplementary Information.
