# Supplementary material for: Hafnium isotope constraints on the nature of the mantle beneath the Southern Lau basin (SW Pacific)
Source: Sci Rep. 2020 Oct 15;10:17476. doi: 10.1038/s41598-020-74565-0 (PMC7566480; doi:10.1038/s41598-020-74565-0)
Supplement: Supplementary file 1 — Supplementary Information. [file 41598_2020_74565_MOESM1_ESM.pdf]

Supplementary information for

Hafnium isotope constraints on the nature of the mantle beneath the Southern Lau basin (SW Pacific)

Quanshu Yan[1,2*], Susanne Straub[3], Paterno Castillo[4],Haitao Zhang[1], Liyan Tian[2,5], Xuefa Shi[1,2]

1, Key Laboratory of Marine Sedimentology and Environmental Geology, First Institute of Oceanography, Ministry of of Natural Resources, Qingdao 266061, China
2, Laboratory for Marine Geology, Qingdao National Laboratory for Marine Science and Technology, Qingdao 266061, China
3, Lamont-Doherty Earth Observatory, Columbia University, Palisades, NY10964, USA
4, Scripps Institution of Oceanography, University of California, San Diego, La Jolla, CA, 92093, USA
5, Institute of Deep-sea Science and Engineering, Chinese Academy of Sciences, Sanya 572000, China
* Correspondence: yanquanshu@163.com or qsyan@fio.org.cn

Table S1 Major element concentrations, trace element ratios, and Hf-Sr-Nd-Pb isotopic compositions of eastern Lau spreading center (ELSC) and Valu Fa ridge (VFR) lavas from the southern Lau basin.

εNd_m refers to the εNd values of the ambient mantle after substracting the contribution from the subduction component. ΔNd refers to the mass fraction of Nd in the mantle wedge that is added by the subduction component. A $\Delta Nd \leq 0$ implies no addition from the subduction component, and, thus, correction is not warranted. The detailed methods for calculating εNdm, ΔNd, ΔεNd P/I and ΔεHfp/I values are given by Pearce et al. [1, 2]. The detailed methods for calculating for εHf* and εNd * (from the addition of total slab fluids) are given by Ribeiro et al. [3]. Major element

concentrations, trace element ratios, and Sr-Nd-Pb isotopic compositions are from Yan et al.[4]. The formulas for calculating these related parameters are presented under Supplementary Dataset Table.

Location	Sample	Lithology	Long.(° W)	Lat.(°S)	Depth(m)	La	Nd	Sm	Yb	Sr	Ba	Nb	Hf	Ta	Pb	Th	U	Th/Ta	Ba/Nb
VFR	L-1	glass	176.71	22.5324	1956	3.84	10.05	3.38	3.25	173.64	102.29	0.97	1.99	0.08	1.63	0.32	0.25	4.0	105.5
VFR	L-3	BL	176.72	22.5322	1887	3.83	9.92	3.34	3.13	182.52	101.58	1.06	2.13	0.09	1.75	0.32	0.26	3.6	95.8
VFR	L-4	glass	176.71	22.5338	1885	3.75	9.56	3.24	3.22	166.6	97.92	0.93	1.91	0.08	1.62	0.31	0.12	3.9	105.3
VFR	L-5	glass	176.72	22.5318	1868	4.2	10.55	3.57	3.42	178.92	103.82	0.8	1.61	0.06	1.59	0.33	0.17	5.5	129.8
VFR	L-6	BL	176.61	22.2161	1776	3.73	8.75	3.03	3.1	160.18	56.25	0.96	1.9	0.08	1.56	0.22	0.06	2.8	58.6
VFR	L-7	BL	176.61	22.2158	1744	5.06	12.65	4.2	3.83	186.58	252.17	1.32	2.59	0.1	2.65	0.42	0.28	4.2	191.0
ELSC	L-2	BL	176.18	20.6701	2228	1.52	5.67	2.05	1.91	99.03	41.13	0.83	1.41	0.07	1.46	0.09	0.19	1.3	49.6
ELSC	L-8	BL	176.24	20.9281	2254	1.64	6.2	2.25	2.51	107.33	53.7	0.9	1.66	0.08	1.38	0.09	0.19	1.1	59.7
ELSC	L-9	BL	176.18	20.6874	2210	5.73	17.05	5.73	5.9	129.67	68.12	3.01	4.21	0.22	1.82	0.35	0.11	1.6	22.6
ELSC	L-10	BL	176.31	20.6804	2201	1.16	3.92	1.45	1.71	97.01	33.84	0.62	0.99	0.05	1.14	0.09	b.d.	1.8	54.6
ELSC	L-11	BL	176.19	20.6854	2249	1.58	5.6	2.02	1.86	92.81	42.03	0.87	1.46	0.07	1.48	0.09	0.2	1.3	48.3
ELSC	L-12	BL	176.18	20.6866	2253	1.69	5.51	2.04	2.35	109.4	40.01	0.86	1.47	0.53	1.28	0.11	b.d.	0.2	46.5

Location	Sample	$^{87}Sr/^{86}Sr$	$^{143}Nd/^{144}Nd$	εNd	$^{206}Pb/^{204}Pb$	$^{207}Pb/^{204}Pb$	$^{208}Pb/^{204}Pb$	$^{176}Hf/^{177}Hf$		εHf	ΔNd	εNdm	ΔεNd P/I	ΔεHf P/I	εNd*	εHf*
								ratios	error(2σ)							
VFR	L-1	0.7033	0.513048	8.00	18.66	15.553	38.342	0.283202	13	15.21	0.267	7.06	1.51	-2.41	9.2	/
VFR	L-3	0.7034	0.513042	7.88	18.612	15.559	38.32	0.283194	15	14.92	0.201	7.18	1.45	-2.31	9.1	/
VFR	L-4	0.7034	0.513051	8.06	18.666	15.55	38.339	0.283195	15	14.95	0.262	7.14	1.28	-2.05	/	/
VFR	L-5	0.7033	0.513049	8.02	18.664	15.556	38.353	0.283198	17	15.08	0.441	6.47	1.41	-2.25	9.1	16.8
VFR	L-6	0.7032	0.513048	8.00	18.457	15.54	38.149	0.283195	11	14.95	0.195	7.31	1.34	-2.15	9.4	16.4
VFR	L-7	0.7033	0.513043	7.90	18.582	15.552	38.249	0.283199	9	15.09	0.237	7.07	1.53	-2.45	8.8	16.1
VFR	L-7†	n.d	n.d	n.d	n.d	n.d	n.d	0.283200	13	/	/	/	/	/	/	/
ELSC	L-2	0.7033	0.513043	7.90	18.43	15.539	38.178	0.283212	14	15.54	0.070	7.65	1.81	-2.90	10.3	17.6
ELSC	L-8	0.7033	0.513042	7.88	18.492	15.548	38.206	0.283201	10	15.14	0.005	7.86	1.58	-2.53	10.0	16.8
ELSC	L-9	0.7032	0.513052	8.08	18.41	15.536	38.135	0.283213	11	15.58	0.077	7.81	1.66	-2.65	8.7	16.2
ELSC	L-10	0.7033	0.513037	7.78	18.462	15.538	38.154	0.283221	17	15.88	0.061	7.57	2.15	-3.43	11.7	19.1
ELSC	L-11	0.7033	0.513041	7.86	18.486	15.54	38.148	0.283219	17	15.81	0.020	7.79	2.02	-3.23	10.2	17.8
ELSC	L-11†	n.d	n.d	n.d	n.d	n.d	n.d	0.283213	15	/	/	/	/	/	/	/
ELSC	L-12	0.7033	0.513038	7.80	18.49	15.549	38.223	0.283216	13	15.69	0.005	7.78	2.01	-3.21	10.2	17.7

mark† represents replicates. n.d, not determined

Formulas for calculating parameters are as follows,

$\varepsilon Nd=((^{143}Nd/^{144}Nd)_{sample}/(^{143}Nd/^{144}Nd)_{CHUR}-1)*10000$, where $(^{143}Nd/^{144}Nd)_{CHUR}=0.512638$

$\varepsilon Hf=((^{176}Hf/^{177}Hf)_{sample}/(^{176}Hf/^{177}Hf)_{CHUR}-1)*10000$, where $(^{176}Hf/^{177}Hf)_{CHUR}=0.282772$

$\Delta Nd=(Nd/Yd-10\hat{}(0.58+1.05\times\log(Hf/Nd)))/(Nd/Yb)$, where Nd, Yb, Hf are concentrations. (Pearce et al., 2007)[3]

$\varepsilon Ndm=\varepsilon Ndw-\Delta Nd\times 3.5$ (Pearce et al., 2007)[3]

$\Delta\varepsilon Nd\ P/I=0.625\times\varepsilon Hf-\varepsilon Nd$. (Pearce et al.,1999, 2007)[2,3]

$\Delta\varepsilon Hf\ P/I=1.6\times\varepsilon Nd-\varepsilon Hf$. (Pearce et al., 1999, 2007)[2,3]

$\varepsilon Nd^*=(((Nd\times{}^{143}Nd/^{144}Nd_{sample})-(13.66\times f_s))/((Hf-(26.64\times f_s)/(1-f_s))\times(1-f_s))/(^{143}Nd/^{144}Nd)_{CHUR}-1)*10000$, where $(^{143}Nd/^{144}Nd)_{CHUR}=0.512638$, f_s=5%(fluid addition), and Nd is concentration. (Ribeiro et al., 2017)[4]

$\varepsilon Hf^*=(((Hf\times{}^{176}Hf/^{177}Hf_{sample})-(0.993\times f_s))/((Hf-(3.51\times f_s)/(1-f_s))\times(1-f_s))/(^{176}Hf/^{177}Hf)_{CHUR}-1)*10000$, where $(^{176}Hf/^{177}Hf)_{CHUR}=0.282772$, f_s=5%(fluid addition), and Hf is concentration. (Ribeiro et al., 2017)[4]

References

1. Pearce, J.A., Kempton, P.D., Nowell, G.M., & Noble, S.R. Hf–Nd element and isotope perspective on the nature and provenance of mantle and subduction components in arc-basin systems: examples from the western Pacific. *J. Petrol*. **40**, 1579–1611 (1999).

2. Pearce, J.A., Kempton, P.D., & Gill, J.B. Hf–Nd evidence for the origin and distribution of mantle domains in the SW Pacific. *Earth Planet. Sci. Lett.* **260**, 98–114 (2007).

3. Ribeiro, J.M., Stern, R.J., Martinez, F., Woodhead, J., Chen, M., & O'hara, Y. Asthenospheric outflow from the shrinking Philippine Sea Plate: Evidence from Hf–Nd isotopes of southern Mariana lavas. *Earth Planet. Sci. Lett*. **478**, 258–271 (2017).

4. Yan, Q., Castillo, P.R., & Shi, X. Geochemistry of basaltic lavas from the southern Lau Basin: input of compositionally variable subduction components. *Int. Geol. Rev.* **54**, 1456–1474 (2012).
